# In Response

**DOI:** 10.4269/ajtmh.2011.10-0617b

**Published:** 2011-02-04

**Authors:** Elizabeth Chizema-Kawesha, John M. Miller, Richard W. Steketee, Victor M. Mukonka, Carlos C. Campbell, Abdirahman Mohamed, Simon Miti

**Affiliations:** E-mail: rsteketee@path.org

Dear Sir:

The success of the malaria control efforts occurring in Zambia^1^ is increasingly evident in other countries in the African region.^2^,^3^ These reports of success similarly are attributable to a mix of integrated and coordinated partner support, intervention delivery, and measurement activities. We agree with Spence-Lewis that acknowledging these successes more broadly is important and several other articles and publications have subsequently recognized this need.^4^–^6^

Furthermore, we agree that improving communication is an enabling factor for success. This is important both centrally to coordinate planning among malaria control partners and peripherally to improve the uptake of interventions in communities and households. The Zambia National Malaria Control Program (ZNMCP) coordinated centrally through the Ministry of Health's National Malaria Control Center has developed a vibrant system of national technical working groups across all the major strategic areas of the malaria program. This is arguably one approach to communication capacity building among those managing national malaria control activities. These groups bring together management and implementing staff within the Ministry of Health and partners and encourage dialogue for a coordinated approach. This approach also encourages collective continuity for strategic priorities in an environment where funding flows and dedicated staffing can be intermittent.

At community level, the ZNMCP supports strategic community leader engagement through focused trainings on malaria, malaria interventions, and the importance of leveraging their leadership positions to control the disease. The ZNMCP also targets focused malaria control messages toward “end-users,” those communities and populations at risk of malaria. These messages include the importance of using interventions, recognizing the symptoms of malaria, getting tested and knowing the test results, and seeking treatment early if infected. These messages are channeled through what we believe are the most effective methods of communicating to populations at risk of malaria and include interpersonal communication, through local health workers, community leaders and community advocates, and mass media channels, especially community radio. Influencing these channels of communication is developing local-level capacity to engage in malaria control activities and contributes to local-level success in malaria control.
